# Supplementary material for: Genome-Wide Identification and Expression Profile Analysis of the NADPH Oxidase Gene Family in Avena sativa L
Source: Int J Mol Sci. 2025 Mar 13;26(6):2576. doi: 10.3390/ijms26062576 (PMC11942474; doi:10.3390/ijms26062576)
Supplement: Supplementary file 1 [file ijms-26-02576-s001.zip › ijms-3438670-supplementary.pdf]

**Table S1.** qRT-PCR primers for *AsRBOHs* gene.

| Primer name | Forward primer (5′-3′) | Reverseprimer(5′-3′) |
|---|---|---|
| 5CG0887860 | GCAACACCATTCATAAGCATTCTAA | TCAGCAACCTCATTCATCACTCC |
| 5AG0846340 | TGCGGTCCAATGCTCATCC | GCTCTTGCGTCCAGTCTCC |
| 5DG0963470 | TTCATCAGCCGCACCACAG | GGAGCCAGAAGTCGTAGAGC |
| 3DG0550720 | TCCCGCCTCCAAATCTTCTTC | AGTGTCTCCAGTTGCCATAGC |
| 3AG0449960 | AGCCACAGCCACCACCAG | GAAGTCGGAGCGGGAGAGG |
| 3CG0504180 | GTGGAGGTGACGCTGGAC | CGGCTGGTTGTGGTGTAGG |
| 5CG0922760 | GACAGCAGCAACACCTTCAG | TCAGCAACCTCATCCATCACC |
| 1DG0159520 | CCTTAGAGCAGACGAGACAACC | GCACCAATTCCTAATCCAACAAGTA |
| 1AG0040750 | TTAAGGAGCAAGCAGAAGAATACG | AACAATCCAGCCATTATCCCAATC |
| 1AG0009010 | CCAAGCAGTTCTCGCAGGAC | GGAGGAGGAGGCAGATGAGG |
| 4CG1304250 | GGCAGAGACGGACAAGAAGG | CGGAGCACCACAGTAGAACAC |
| 3CG0504790 | CGACGCTCCACACGCTTG | CACGACGCTACGCCTGAAG |
| 3AG0450850 | CACCAGGACTACGGACTTCATTC | GCGGCAGACAGGGAGGAG |
| 3DG0551470 | TCACCAGGACCACGGACTTC | GGCAGACAGGGAGGAGGATG |
| 4DG0724710 | TCGCACCACTTCTTCGTCATC | TGTCCGCTCTTGTATCTGAACC |
| 4AG0580180 | CAGGACTACAAGCAATACGACATC | GACGCCACGGAACCACTC |
| 1AG0015390 | CACATAATGTCGATCTCCTTCA | CACGCCACGGCAACCACTG |
| 1AG0047560 | CCTCATAATGTCGTTCTCCTTCAC | CGCTCACTCCATAATTCTTCTCG |
| 1DG0166070 | TACTCATACGCTTGCTCACCTG | TTCTCCTGGAACTTGTCACTTGG |
| 5CG0916260 | TCAACGACAACATCAACTTCCAC | CCGCACGAACCACCAGTAG |
| 5AG0820170 | CGCCACTCCGCTCATCAG | CTCCGCCACCTCGTTCATC |
| 5DG0988170 | CGGTTCAAGGAGGAGAAGGAG | CGACGACGCTCTGGATGG |
| 3CG0479430 | GCGGTGGGTTTGATGGTCTG | CTTGCTTCGGTCGTATCTCTTCC |
| 5DG0959800 | CAGAGGAGGTTAAGGAGATTATTGC | CAGTAATGGCGAAGTGGATTGG |
| 5AG0850070 | GAGAGGAGGTTAAGGAGATATTG | AGTAATGGCGAAGTGGAATGG |
| 1AG0047060 | CCACCTCTTCGTCATCGTCTAC | GCCGTCTTCTTCTGCCACTC |
| 1AG0047090 | CCACCTCTTCATCGTCGTCTAC | GTGGTCTTCTGCTGCCACTTA |
| 1DG0165660 | ACCTCTTCATCGTCGTCTAC | TCGTCTTCTCGTGCCACTAG |
| 1DG0165630 | CCACTCTTCATCGCGTCTAA | CTCGTCTTCTCCTGCCACTGG |
| 1AG0014930 | CACCTCTTCGTCGTCGTCTAC | GTCGTCTTCTTCTGCCACTCG |
| 3DG0531950 | GAGAGGAGGTTAAGGAGATTATTGC | CAGTAATGGCGAAGTGGATTG |
| 3AG0430660 | ATCCAGAAGGTTGCGGTATATCC | CCTCAGAGAAGACAGTCCTAAGC |
| 7DG1382190 | CCTGCTCATCGTCGTCTACC | GGCTCGTAATGTTCGTTCACC |
| 7AG1225850 | CTGCTCATCGTCGTCTACT | GCTCGTAATGTTCGTTCACA |
| UnG1437940 | ACTGCTCATGGTCGTCTACG | GGCTCGTAATGTCGTTCACG |
